# Suppressed renoprotective purines in COVID-19 patients with acute kidney injury

**DOI:** 10.1038/s41598-022-22349-z

**Published:** 2022-10-17

**Authors:** Edwin K. Jackson, Georgios D. Kitsios, Michael Y. Lu, Caitlin M. Schaefer, Cathy J. Kessinger, Bryan J. McVerry, Alison Morris, Bernard J. C. Macatangay

**Affiliations:** 1 Department of Pharmacology and Chemical Biology, University of Pittsburgh School of Medicine, 100 Technology Drive, Room 514, Pittsburgh, PA 15219 USA; 2 Department of Medicine, Division of Pulmonary, Allergy and Critical Care Medicine, University of Pittsburgh School of Medicine, Pittsburgh, PA USA; 3 Department of Medicine, Division of Infectious Disease, University of Pittsburgh School of Medicine, Pittsburgh, PA USA

**Keywords:** Infectious diseases, Kidney diseases

## Abstract

Acute kidney injury (AKI) is common in patients hospitalized for COVID-19, complicating their clinical course and contributing to worse outcomes. Animal studies show that adenosine, inosine and guanosine protect the kidney against some types of AKI. However, until now there was no evidence in patients supporting the possibility that abnormally low kidney levels of adenosine, inosine and guanosine contribute to AKI. Here, we addressed the question as to whether these renoprotective purines are altered in the urine of COVID-19 patients with AKI. Purines were measured by employing ultra-high-performance liquid chromatography-tandem mass spectrometry with stable-isotope-labeled internal standards for each purine of interest. Compared with COVID-19 patients without AKI (n = 23), COVID-19 patients with AKI (n = 20) had significantly lower urine levels of adenosine (*P* < 0.0001), inosine (*P* = 0.0008), and guanosine (*P* = 0.0008) (medians reduced by 85%, 48% and 61%, respectively) and lower levels (*P* = 0.0003; median reduced by 67%) of the 2nd messenger for A_2A_ and A_2B_ adenosine receptors, i.e., 3’,5’-cAMP. Moreover, in COVID-19 patients with AKI, urine levels of 8-aminoguanine (endogenous inhibitor of inosine and guanosine metabolism) were nearly abolished (P < 0.0001). In contrast, the “upstream” precursors of renoprotective purines, namely 5’-AMP and 5’-GMP, were not significantly altered in COVID-19 patients with AKI, suggesting defective conversion of these precursors by CD73 (converts 5’-AMP to adenosine and 5’-GMP to guanosine). These findings imply that an imbalance in renoprotective purines may contribute to AKI in COVID-19 patients and that pharmacotherapy targeted to restore levels of renoprotective purines may attenuate the risk of AKI in susceptible patients with COVID-19.

## Introduction

In COVID-19 patients, acute kidney injury (AKI) is common and is associated with in-hospital mortality^1^ and long-term kidney dysfunction in patients who recover from COVID-19^2^. Greater than 20% of hospitalized COVID-19 patients and more than 50% of COVID-19 patients in the intensive care unit (ICU) develop AKI^3^. AKI in ICU COVID-19 patients markedly increases the risk for need of renal replacement therapy^3^, and up to 50% of COVID-19 patients in the ICU who develop AKI do not survive^4^.

Specific treatments to prevent or attenuate COVID-19-associated AKI do not exist. To a large extent this unmet medical need is likely due to the fact the pathophysiology of AKI in COVID-19 patients is multifactorial^3–5^. Proposed mechanisms of COVID-19-associated AKI include^4^: (1) direct viral toxicity in podocytes and proximal tubular epithelial cells; (2) lung-kidney interactions; (3) heart-kidney interactions; (4) cytokine storm; (5) rhabdomyolysis; (6) endotheliitis; (7) coagulation disturbances leading to thrombotic microangiopathy; (8) sepsis; and (9) ischemia–reperfusion injury. Since these proposed mechanisms are not mutually exclusive, likely there is considerable patient-to-patient variability and within patient variability over time in the particular combination of pathophysiological mechanisms that culminate in AKI. Thus, COVID-19-associated AKI is both complex and dynamic; a situation that does not bode well for a strategy focused on a specific pathophysiological mechanism.

Overlooked among the potential contributing factors to COVID-19-associated AKI is the possibility that key biochemical pathways that protect against AKI (herein referred to as “renoprotective”) are dysfunctional in COVID-19, thus increasing the patient’s susceptibility to any given complex of triggering pathophysiological mechanisms. Elucidation of such dysfunctional renoprotective mechanisms could lead to treatments that boost the overall resistance of the kidney to injurious stimuli and thereby could offer benefit to a large percentage of COVID-19 patients regardless of AKI pathophysiology.

In recent decades it has become clear that adenosine, inosine, and guanosine can protect the kidney against AKI induced by several types of noxious stimuli. In this regard, adenosine mediates protection against ischemia–reperfusion (I/R) induced AKI via activating A_1_, A_2A_ and A_2B_ receptors^6^; and A_3_ adenosine receptors afford protection against sepsis-induced AKI^7^. As shown by Lee and coworkers, A_1_ knockout mice and wild-type mice treated with a selective A_1_ blocker are more susceptible to I/R-induced AKI; whereas, in wild-type mice a selective A_1_ agonist protects against I/R-induced AKI^8^. Moreover, selective intrarenal A_1_ receptor overexpression protects the kidney from AKI induced by liver-kidney crosstalk^9^. The mechanisms by which A_1_ receptors protect the kidney include reducing metabolic demand, necrosis, apoptosis and inflammation and increasing the production of IL-11^10^.

As reviewed by Okusa^11^ and Vincent and Okusa^12^, A_2A_ receptors also protect kidneys against I/R-induced AKI. The mechanisms by which A_2A_ receptors protect the kidney involve inhibition of neutrophil adhesion and the production of reactive oxygen species^13^ and prevention of dendritic cells from activating natural killer cells to produce interferon-γ^14^. The renoprotective effects of A_2A_ receptors are enhanced by blockade of phosphodiesterase 4, indicating a critical role for 3’,5’-cAMP in renoprotection induced by activation of A_2A_ receptors^15^.

Renovascular A_2B_ and A_3_ receptors also appear to be renoprotective. For example, Eltzschig and coworkers reported that A_2B_ receptors mediate renoprotection induced by ischemic preconditioning by suppressing inflammation and the production of reactive oxygen species and N-derived oxidants^16^. Although the role of A_3_ receptors in the kidney is less well studied, Lee and colleagues demonstrated that A_3_ activation affords renoprotection against sepsis-induced AKI by attenuating the inflammatory response to sepsis^7^. The renoprotective roles of adenosine are further supported by the facts that CD39 (converts ATP to ADP and ADP to 5’-AMP) and CD73 (metabolizes 5’-AMP to adenosine) protect the kidney from I/R injury^17–20^.

Adenosine, however, is not the only renoprotective purine. Mόdis and coworkers developed an in vitro model of acute tubular necrosis and screened 1280 pharmacologically active compounds for cytoprotection^21^. Their most important findings were that both adenosine and inosine afforded cytoprotection that was mediated by A_3_ receptors. Moreover, studies by Kelly and coworkers demonstrate that guanosine supplementation protects against I/R-induced AKI^22^.

Together, the extant literature suggests that adenosine, inosine and guanosine comprise a trio of purines that protect the kidney from a variety of injurious stimuli. We hypothesize that this renoprotective purinergic system is compromised in some COVID-19 patients leading to more severe kidney damage in such patients in response to various triggering pathophysiological pathways. If this hypothesis is correct, restoring renoprotective purines with pharmacotherapy may improve outcomes in COVID-19 patients. This concept is supported by the recent findings that inhalation of an adenosine aerosol improves oxygenation and lung radiological characteristics and reduces mortality in severe COVID-19 pneumonia^23^. Here, we show that the concentrations of all three renoprotective purines in urine are severely suppressed in COVID-19 patients with AKI compared with COVID-19 patients with normal renal function.

## Methods

### Clinical study design

We conducted a nested case–control study of hospitalized patients with COVID-19 pneumonia based on diagnosis of AKI (n = 23) or no AKI (n = 21) at the time of enrollment. These patients had been enrolled in two prospective, observational cohort studies at the University of Pittsburgh Medical Center (UPMC) from August 2020 through March 2021^24^. Inclusion criteria included a documented SARS-CoV-2 infection in adults (age 18–90 years) admitted in dedicated hospital wards for COVID-19 or the intensive care unit (ICU). Exclusion criteria were inability to obtain consent, prisoner status, comfort-measures-only status, and blood hemoglobin < 8 g/dL. The University of Pittsburgh Institutional Review Board approved the studies (STUDY19050099 and STUDY20040036) and written informed consent was provided by all participants or their surrogates in accordance with the Declaration of Helsinki. We recorded clinical characteristics, including demographics, laboratory data, vital signs, respiratory support, and clinical outcomes. AKI was defined according to RIFLE criteria^25^.

### Biospecimen collection and processing

From enrolled subjects, we collected blood samples for centrifugation and separation of plasma on the date of enrollment. We also collected urine in a specimen cup via an indwelling bladder catheter or via normal urethral voiding. Urine samples for analysis of purines were obtained synchronously with the diagnosis of AKI. Collected samples were rapidly frozen at – 80 °C until analyzed for purines. One urine sample in the AKI group was severely contaminated with hemolyzed blood which precluded measurement of urinary purines in that sample.

### Analysis of urinary purines

COVID-19 urine samples were diluted 30-fold with distilled water so that the levels of urinary purines in the diluted samples were within the range of the standard curves, and a mix of stable-isotope internal standards (see Table 1) was added to the diluted samples. Viral particles in samples were inactivated by heating to 100 °C for 90 s^26^.

**Table 1:** Summary of UPLC-MS/MS assays.

Internal standard or Target purine	Vendor	Precursor ion (m/z)	Collision energy (volts)	Product ion (m/z)	Approximate retention time (min)
**Analysis of adenosine**					
^13^C_10_-Adenosine	Medical Isotopes, Inc.	278	19	141	3.18
Adenosine	Sigma-Aldrich	268	19	136	3.18
**Analysis of 3’,5’-cAMP**					
^13^C_5_-3’,5’-cAMP	Toronto Research Chemicals	335	28	136	3.23
3’,5’-cAMP	Sigma-Aldrich	330	28	136	3.23
**Analysis of 5’-AMP**					
^13^C_10_-5’-AMP	Medical Isotopes, Inc.	358	19	141	1.60
5’-AMP	Sigma-Aldrich	348	19	136	1.60
**Analysis of inosine**					
^15^N_4_-Inosine	Cambridge Isotope Laboratories	273	20	141	2.92
Inosine	Sigma-Aldrich	269	20	137	2.92
**Analysis of hypoxanthine**					
^13^C_5_-Hypoxanthine	Cambridge Isotope Laboratories	141.8	22	124	1.77
Hypoxanthine	Sigma-Aldrich	136.8	22	119	1.77
**Analysis of xanthine**					
^15^N_2_-Xanthine	Cambridge Isotope Laboratories	154.9	20	137.8	1.89
Xanthine	Sigma-Aldrich	152.9	20	135.8	1.89
**Analysis of guanosine**					
^13^C_10_,^15^N_5_-Guanosine	Medical Isotopes, Inc.	299	20	162	2.94
Guanosine	Sigma-Aldrich	284	20	152	2.94
**Analysis of 3’,5’-cGMP**					
^13^C_5_-3’,5’-cGMP	Toronto Research Chemicals	351	16	152	3.23
3’,5’-cGMP	Sigma-Aldrich	346	16	152	3.23
**Analysis of 5’-GMP**					
^13^C_10_-5’-GMP	Medical Isotopes, Inc.	374	15	157	1.60
5’-GMP	Sigma-Aldrich	364	15	152	1.60
**Analysis of guanine**					
^13^C_2_,^15^N-Guanine	Medical Isotopes, Inc.	155	20	138	1.48
Guanine	Sigma-Aldrich	152	20	135	1.48
**Analysis of 8-aminoguanine**					
^13^C_2_,^15^N-8-Aminoguanine	Medical Isotopes, Inc.	170	18	153	1.43
8-Aminoguanine	Toronto Research Chemicals	167	18	150	1.43

Purines in urine were measured as previously described^27^ using ultra-high-performance liquid chromatography-tandem mass spectrometry (UPLC-MS/MS). Samples were injected into a Waters Acquity UPLC system (ThermoFisher Scientific, San Jose, CA) and purines were resolved using a Waters BEH C18 column (1.7 µm beads; 2.1 × 150 mm; Milford, MA) and assayed using a TSQ Quantum triple quadrupole mass spectrometer (ThermoFisher Scientific) operating in the multiple reaction monitoring mode with a heated electrospray ionization source. The mobile phase consisted of a linear gradient of two buffers: Buffer A, 1% acetic acid in water; Buffer B, methanol. The mobile phase flow rate was 300 μL/min. The gradient (A/B) was: from 0 to 2 min, 99.6%/0.4%; from 2 to 3 min, to 98.0%/2.0%; from 3 to 4 min, to 85.0%/15.0%; from 4 to 6.5 min; to 99.6%/0.4%. Instrument settings were: sample tray temperature, 10 °C; column temperature, 50 °C; ion spray voltage, 4.0 kilovolts; ion transfer tube temperature, 350 °C; source vaporization temperature, 320 °C; Q2 CID gas, argon at 1.5 mTorr; sheath gas, nitrogen at 60 psi; auxillary gas, nitrogen at 35 psi; Q1/Q3 width: 0.7/0.7 units full-width half-maximum; scan width, 0.6 units; scan time, 0.01 s. Precursor ions, product ions, retention times and collision energies are provided in Table 1.

### Analysis of urinary creatinine

Urine samples were assayed for creatinine levels using the Creatinine Colorimetric Assay Kit (No. 500701; Cayman Chemical, Ann Arbor, MI).

### Analysis of plasma biomarkers

Plasma samples were available from 38 of the 43 patients for analysis of biomarkers. These were profiled with a custom Luminex multi-analyte panel (R&D Systems, Minneapolis) targeting biomarkers associated with pneumonia outcomes including receptor for advanced glycation endproducts (RAGE), interleukin (IL)-6, IL-8, IL-10, tumor necrosis factor receptor 1 (TNFR1), soluble interleukin 1 receptor-like 1 (ST2), fractalkine, angiopoietin-2, procalcitonin and pentraxin-3, as previously described^28^.

### Statistical methods

**Figure 1:**
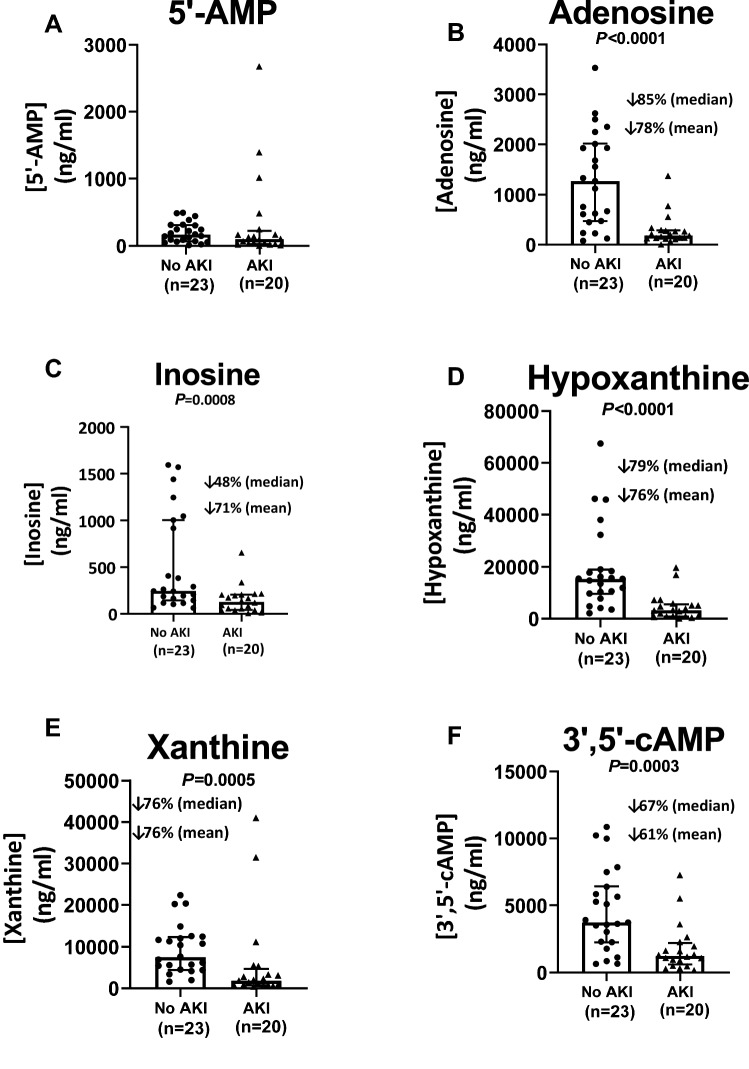
Effects of acute kidney injury (AKI) on urine levels (ng/ml) of (**A**) 5’-AMP, (**B**) adenosine, (**C**) inosine, (**D**) hypoxanthine, (**E**) xanthine and (**F**) 3’,5’-cAMP. Dot plots show individual values. Also shown are medians with interquartile ranges and the percentage decreases (↓%) in both the median and mean values in AKI compared to patients without AKI (No AKI).

**Figure 2:**
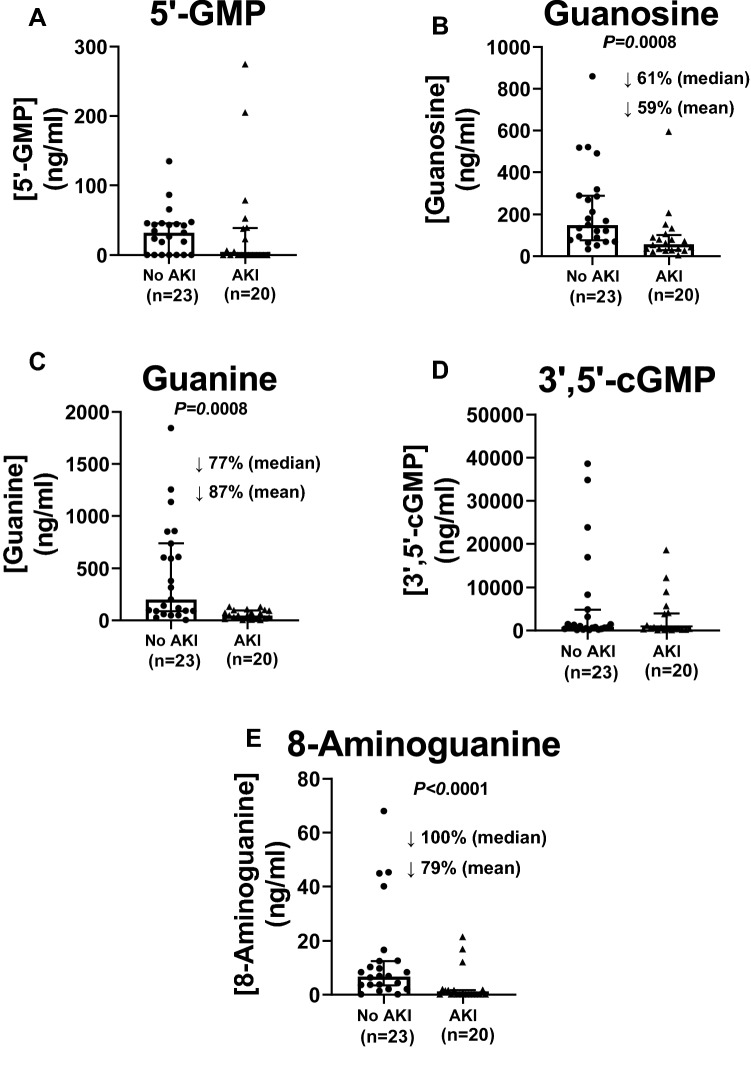
Effects of acute kidney injury (AKI) on urine levels (ng/ml) of (**A**) 5’-GMP, (**B**) guanosine, (**C**) guanine, (**D**) 3’,5’-cGMP and (**E**) 8-aminoguanine. Dot plots show individual values. Also shown are medians with interquartile ranges and the percentage decreases (↓%) in both the median and mean values in AKI compared to patients without AKI (No AKI).

Tests for normality and homoscedasticity indicated that values for urinary purines were not normally distributed and that variances between the two groups (No AKI versus AKI) were unequal. Therefore, the data were transformed using the Box-Cox method and retested for normality and homoscedasticity. Transformed data passed tests for normality and homoscedasticity; therefore, the transformed data were statistically compared in COVID-19 patients without versus with AKI using an unpaired, 2-tailed, Student’s t-test. Dot plots of urinary purine data in Figs. 1 and 2 show median values with interquartile ranges. Also shown in these figures are the percentage reductions in AKI patients of the median urinary purine values. Creatinine clearances were estimated by the following standard equation^29^: creatinine clearance (ml/min) = concentration of creatinine in urine (mg/dl) times the urine excretion rate (ml/min) divided by the concentration of creatinine in plasma (mg/dl). The excretion rate of purines (ng/min) was estimated by multiplying the concentration of purines in urine (ng/ml) by the urine excretion rate (ml/min). Correlations between purine excretion rates and creatinine clearances or plasma biomarkers were assessed using the nonparametric Spearman’s rank correlation coefficient method. Baseline characteristics and outcomes between groups were compared using Wilcoxon tests for continuous variables and Fisher’s exact test for categorical variables. The criterion for significance was *P* ≤ 0.05.

### Ethical approval

The University of Pittsburgh Institutional Review Board approved the studies (STUDY19050099 and STUDY20040036).

### Informed consent

Written informed consent was provided by all participants or their surrogates in accordance with the Declaration of Helsinki.

## Results

As shown in Table 2, with the exception of age, demographics were similar in patients with AKI versus those without AKI. In both groups, measures of disease severity, other than AKI, were similar. For example, at the time of urine collection the two groups were similar with respect to the percentage of patients in the ICU, intubated or on ECMO (Table 2). Also, patients with or without AKI had similar scores on the WHO Ordinal Scale of Severity (Table 2). As expected, AKI patients had significantly elevated levels of blood urea nitrogen and plasma creatinine (Table 3). AKI patients also had higher concentrations of inflammatory biomarkers (angiopoietin-2, interleukin-8, procalcitonin, ST2, fractalkine, pentraxin-3, RAGE and TNFR-1) (Table 3). The cause of AKI was either acute tubular necrosis (9 patients), cardiorenal AKI (3 patients) or pre-renal AKI (8 patients). Table 4 summarizes patient outcomes and demonstrates significantly higher 30-mortality and a near significantly (*P* = 0.08) higher 90-day mortality in AKI patients.

**Table 2:** Summary of patient demographics, history and disease severity.

	No AKI	AKI	P-value
Sample size	23	21	
Age (years)	58.0^a^ [42.2, 64.6]^b^	67.0^a^ [62.0, 73.2]^b^	0.02
Body mass index (kg/m^2^)	31.8^a^ [29.5, 39.3]^b^	29.7^a^ [27.1, 34.7]^b^	0.2
Male (%)	13 (56.5)	14 (66.7)	0.7
**Race**			
African American (%)	6 (26.1)	5 (23.8)	0.32
Caucasian (%)	17 (73.9)	14 (66.7)	
Not specified (%)	0 (0.0)	2 (9.5)	
History of COPD (%)	1 (4.3)	3 (14.3)	0.53
History of immunosuppression (%)	2 (8.7)	4 (19.0)	0.58
History of CRF (%)	1 (4.3)	2 (9.5)	0.93
History of alcohol (%)	23 (100.0)	21 (100.0)	1.0
Active neoplasm (%)	1 (4.3)	1 (4.8)	1.0
ICU patient (%)	17 (73.9)	15 (71.4)	1.0
WHO Ordinal Scale of Severity	6.0^a^ [5.0, 7.5]^b^	6.0^a^ [5.0, 7.0]^b^	0.83
Intubated (%)	11 (47.8)	7 (33.3)	0.5
Veno-Venous ECMO (%)	4 (33.3)	0 (0.0)	0.12

COPD, chronic obstructive pulmonary disease; CRF, chronic renal failure; ECMO, extracorporeal membrane oxygenation; ICU, intensive care unit. WHO Ordinal Scale of Severity includes metrics of respiratory support, vasopressors and ECMO.

^a^Median.

^b^Interquartile range.

**Table 3:** Summary of blood chemistry and biomarkers of inflammation.

	No AKI	AKI	P-value
Sample size	23	21 (15^a^)	
Blood urea nitrogen (mg/dL)	18.0 [12.5, 28.0]	66.0 [40.0, 100.0]	< 0.01
Creatinine (mg/dL)	0.7 [0.6, 0.8]	2.5 [1.7, 3.3]	< 0.01
White blood cell count (1000/µL)	9.7 [5.7, 12.8]	7.6 [6.1, 15.1]	0.99
Hemoglobin (g/dL)	12.9 [12.1, 14.2]	11.1 [9.4, 13.4]	< 0.01
Platelets (1000/µL)	207.0 [161.0, 270.0]	163.0 [142.0, 202.0]	0.21
CO_2_ (mmol/L)	28.0 [24.0, 30.0]	23.0 [21.0, 27.0]	< 0.05
Angiopoietin-2 (pg/mL)	5490.8 [2173.8, 15,011.9]	28,035.4 [3193.4, 41,064.9]	< 0.05
Interleukin-8 (pg/mL)	11.2 [7.3, 20.5]	26.2 [18.3, 32.2]	< 0.05
Interleukin-6 (pg/mL)	21.7 [6.9, 37.8]	82.6 [13.2, 114.8]	0.16
Procalcitonin (pg/mL)	102.3 [67.9, 161.5]	1039.5 [289.5, 2547.2]	< 0.01
ST2 (pg/mL)	117,938.8 [61255.1, 164,618.7]	258,805.9 [188349.4, 433,301.3]	< 0.01
Fractalkine (pg/mL)	187.2 [185.1, 976.9]	3264.7 [2024.9, 5311.8]	< 0.01
Interleukin-10 (pg/mL)	0.7 [0.6, 4.4]	0.6 [0.6, 9.9]	0.58
Pentraxin-3 (pg/mL)	6338.2 [2024.9, 11,203.5]	19,529.0 [11424.0, 25,708.1]	< 0.01
RAGE (pg/mL)	12,837.2 [2567.6, 33,369.6]	66,377.2 [7225.7, 124,459.9]	< 0.05
TNFR-1 (pg/mL)	2409.6 [1792.9, 3598.6]	9297.0 [6966.2, 17,229.2]	< 0.01

^a^Sample size for plasma biomarkers using Luminex analysis was 15. Values are median and interquartile range.

**Table 4:** Patient outcomes.

	No AKI	AKI	P-value
Sample size	23	21	
**30-day mortality**			
Survived (%)	19 (82.6)	10 (47.6)	< 0.05
Died (%)	4 (17.4)	11 (52.4)	
**90-day mortality**			
Survived (%)	17 (73.9)	10 (47.6)	0.08
Died (%)	6 (26.1)	11 (52.4)	
**Discharged to:**			
Home (%)	9 (52.9)	4 (40.0)	0.5
Hospitalization (%)	1 (5.9)	0 (0.0)	
Long term acute care (%)	3 (17.6)	1 (10.0)	
Skilled nursing facility (%)	4 (23.5)	5 (50.0)	

Although urine levels of 5’-AMP, the main precursor of adenosine biosynthesis, were not suppressed in AKI patients (Fig. 1A), median urine levels of adenosine were reduced in AKI patients by 85% (Fig. 1B). Consistent with reduced adenosine biosynthesis, urine levels of adenosine’s “downstream” metabolites were likewise reduced. In this regard, median urine levels of inosine, hypoxanthine and xanthine were depressed by 48%, 79% and 76%, respectively (Fig. 1C–E). Notably, urine levels of 3’,5’-cAMP, a key second messenger involved in A_2A_- and A_2B_-receptor signaling following binding of adenosine, were also reduced (Fig. 1F).

Although variable, urine levels of 5’-GMP, the main precursor of guanosine, were not reduced (Fig. 2A) in AKI patients; yet, in AKI patients median urine levels of guanosine (Fig. 2B) and its metabolite guanine (Fig. 2C) were decreased by 61% and 77%, respectively. Unlike 3’,5’-cAMP, the urine levels of 3’,5’-cGMP were not reduced in AKI patients (Fig. 2D).

Urine levels of 8-aminoguanine, an endogenous inhibitor of purine nucleoside phosphorylase (PNPase) that induces natriuresis and protects against salt-induced hypertension^30,31^, were undetectable in most AKI patients (Fig. 2E). Since inhibition of PNPase increases urine levels of PNPase substrates^32^, the reduction in 8-aminoguanine levels is consistent with decreases in urine levels of inosine and guanosine.

**Figure 3:**
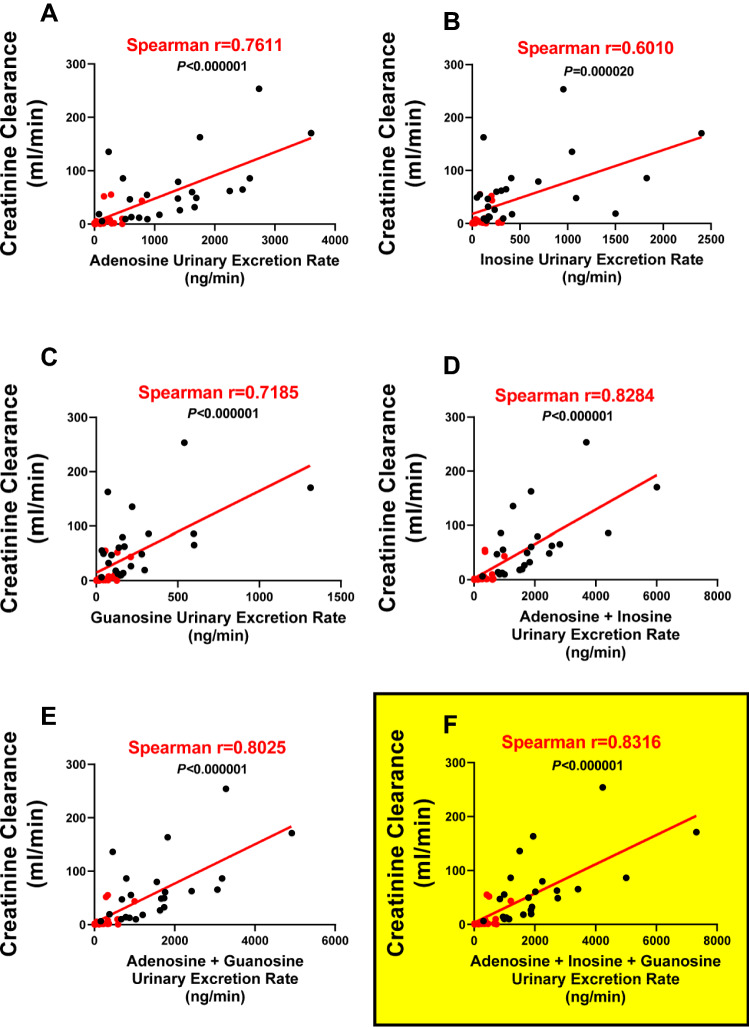
Correlations between urinary excretion rates (ng/min) of adenosine (**A**), inosine (**B**), guanosine (**C**), adenosine + inosine (**D**), adenosine + guanosine (**E**) and adenosine + inosine + guanosine (**F**) versus creatinine clearance. The summation of the excretion rates of adenosine + inosine + guanosine (**F**) yielded the strongest positive correlation with renal function. Red symbols represent data from patients with acute kidney injury.

We also evaluated whether concentrations of the different renoprotective purines alone or in combination were associated with glomerular filtration rate (GFR) as estimated by creatinine clearance (Fig. 3). As shown, individually all three renoprotective purines were significantly and positively correlated with creatinine clearance. However, the strongest correlation was the summation of the urine concentrations of adenosine + inosine + guanosine (Spearman r coefficient, 0.8316; *P* < 0.000001; Fig. 3F). The summation of the urine concentrations of adenosine + inosine + guanosine was also significantly, yet inversely, correlated with the plasma inflammatory biomarkers interleukin-8, procalcitonin, ST2, fractalkine and TNFR-1 (Spearman r coefficients: − 0.3775, − 0.5492, − 0.4981, − 0.4973 and − 0.6197, respectively; P-values: 0.0213, 0.0004, 0.0017, 0.0017 and < 0.0001, respectively). The summation of the urine concentrations of adenosine + inosine + guanosine was also near significantly and inversely correlated with the plasma inflammatory biomarkers pentraxin-3 and RAGE (Spearman r coefficients: − 0.3186 and − 0.2876, respectively; P-values: 0.0546 and 0.0843, respectively).

## Discussion

Here we show that the urine concentrations of three known renoprotective purines, namely adenosine, inosine and guanosine, were severely depressed in COVID-19 patients with AKI compared with COVID-19 patients without AKI. Moreover, the urinary excretion rates of these renoprotective purines were strongly correlated with renal outcomes in COVID-19 patients. The strongest correlation (Spearman r coefficient, 0.8316; *P* < 0.000001) was between the sum of the urinary excretion rates of adenosine + inosine + guanosine versus renal function as assessed by creatinine clearance. Although correlation per se does not prove causation, given the known acute renoprotective effects of adenosine, inosine and guanosine, these data suggest that lack of exposure of the kidneys to renoprotective purines may contribute to poor renal outcomes in COVID-19 patients.

The classical pathway of extracellular production of adenosine is via CD73, an ectoenzyme that converts extracellular 5’-AMP to extracellular adenosine^33^. Importantly, CD73 also metabolizes other ribonucleoside 5’-monophosphates, for example 5’-GMP, to their corresponding nucleosides, albeit with lower efficiency compared to 5’-AMP^34^. Because CD73 mediates the last step in the formation of extracellular adenosine and guanosine, either the concentrations of extracellular 5’-AMP and 5’-GMP or the activity of CD73 would be rate limiting with respect to formation of extracellular adenosine (and its downstream metabolite inosine) and guanosine. Therefore, lower urine concentrations of adenosine, inosine and guanosine could be due to either reduced concentrations of extracellular CD73 substrates (i.e., 5’-AMP and 5’-GMP) or diminished expression of CD73 in some cellular compartments. As shown here, urine levels of 5’-AMP and 5’-GMP did not differ significantly between patients with AKI versus patients without AKI suggesting that reduced CD73 expression may be the underlying cause of diminished urine levels of renoprotective purines in COVID-19 AKI patients. Although this implication requires direct confirmation of reduced CD73 expression in COVID-19 patients with AKI and although it is unknown which tissues/cells lack sufficient CD73 in COVID-19 patients with AKI, it is notable that CD73 expression on CD8 + T, natural killer T and natural killer cells is reduced in patients with COVID-19^35^. Moreover, recent findings by us^36^ and others^37^ demonstrate reduced numbers of CD73-expressing T cells in persons living with HIV (PLWH). Thus, it is conceivable that viral suppression of extracellular purine levels is a general mechanism that contributes to poor outcomes in patients with systemic viral infections. It is also possible that, in addition to COVID-19 patients and PLWH, suppression of extracellular purine levels is a more general mechanism that increases the risk of AKI in a broad spectrum of patients with severe illnesses.

In the present study, we assessed overall exposure of the kidneys to renoprotective purines by measuring the urine concentrations and urinary excretion rates of these purines. Likely, purines in the urine derive from both renal production and renal filtration of plasma purines. Miller and coworkers conducted stop-flow experiments in dogs subjected to renal ischemia and concluded that adenosine and inosine in the urine are derived, at least in part, from ecto-5’-nucleotidase (now known to be CD73) in the proximal tubules^38^. Thompson et al. explored in dogs the fate of plasma adenosine using radiolabeled adenosine as a tracer^39^. Their studies suggested that plasma adenosine also contributes to the urinary excretion of adenosine. Therefore, likely purines in the urine derive from both filtration of circulating purines and production of purines by renal epithelial cells, and likely both pools of renoprotective purines can protect against AKI.

Because adenosine^17^, inosine^40–42^ and guanosine^43–45^ are anti-inflammatory purines, we hypothesize a causal link between low levels of these purines and inflammation in COVID-19 patients with AKI. Indeed, our results show that in COVID-19 patients with poor renal outcomes, extracellular anti-inflammatory purines are reduced while inflammatory cytokines are significantly elevated. In further support of this hypothesis, our findings show that plasma levels of interleukin-8, procalcitonin, ST2, fractalkine and TNFR-1 (biomarkers of inflammation and pneumonia) are inversely correlated with the levels of anti-inflammatory purines (adenosine + inosine + guanosine) in the urine. Again, there is a notable parallel between PLWH and COVID-19 patients with AKI. In this regard, CD73 deficiency in PLWH may contribute to a pro-inflammatory state that contributes to adverse cardiovascular outcomes^46,47^; and in COVID-19 patients reduced levels of CD73-derived purines correlate with a pro-inflammatory state that may contribute to AKI.

8-Aminoguanine is a naturally-occurring inhibitor of PNPase^48^. PNPase metabolizes inosine and guanosine^49^, and recent evidence suggests that PNPase may also metabolize adenosine under some circumstances^50^. Therefore, it is conceivable that the deficiency of 8-aminoguanine in COVID-19 patients with AKI also contributes to the deficiency of renoprotective purines.

Studies by Correale et al. show that inhalation of an adenosine aerosol improves oxygenation and lung radiological characteristics and reduces mortality in severe COVID-19 pneumonia^23^. However, the direct administration of adenosine intravenously to provide renoprotection would be problematic due to the adverse effects of systemically administered adenosine^51^. An implication of the current findings is that purine nucleoside-elevating drugs may be beneficial with regard to improving renal outcomes, and possibly outcomes in general, in seriously ill COVID-19 patients. Our recent finding that dipyridamole reduces immune activation in antiretroviral-treated PLWH^46,47^ further supports this notion. Dipyridamole blocks equilibrative-nucleoside transporters (ENTs) and thereby elevates the extracellular levels of anti-inflammatory purine nucleosides^52,53^.

Another possible approach to augment extracellular anti-inflammatory purine nucleosides is the utilization of prolyl hydroxylase (PHD) inhibitors. As reviewed by Eltzschig and colleagues^54^, in the presence of oxygen PHDs hydroxylate hypoxia-inducible factor-α (HIF-α), a process that promotes proteosome-mediated degradation of HIF-α. In hypoxic microenvironments, HIF-α accumulates and dimerizes with HIF-1β. The HIF-α/HIF-1β dimer is a transcription factor that increases the expression of CD73, A_2A_ receptors and A_2B_ receptors and decreases the expression of ENTs and adenosine kinase (an enzyme that decreases adenosine levels)^54^. Thus, PHD inhibition would be expected to increase not only extracellular levels of adenosine, but also adenosine’s cognate receptors. The concept that PHD inhibitors may attenuate AKI in patients with COVID-19 is further supported by a recent report that roxadustat, a potent PHD inhibitor, protects against I/R-induced AKI in mice^55^. The relative role of purines versus erythropoietin in the renoprotective effects of PHD inhibitors requires additional studies.

There are several limitations of the current study. We limited the scope of our investigation to answer the following question: *Is there evidence that the suppression of renoprotective purines contributes to AKI in patients with COVID-19?* To address this question, we compared renoprotective purine levels in the urine of patients with COVID-19 who do not have AKI versus patients with COVID-19 who do have AKI. However, the current study does not address whether renoprotective purines are suppressed in AKI associated with other disease states; nor do our findings show that the Sars-CoV-2 virus directly suppresses renoprotective purines. Indeed, May and colleagues recently completed a comprehensive, multi-center retrospective cohort study that examined 284 kidney biopsies from COVID-19 patients and did not detect direct kidney infection by SARS-CoV-2^56^. What we can conclude is that, by whatever mechanism, exposure of kidneys to renoprotective purines is suppressed in COVID-19 patients with AKI.

What is the value of our findings? There are at least 3 important aspects of our results that add value. First, our results provide a rationale for conducting clinical trials to examine whether upregulating renoprotective purines reduces the burden of AKI in COVID-19 patients. Second, our results suggest an approach to optimize the design of such clinical trials. By using low urine levels of renoprotective purines as an inclusion criterion, clinical trials would be enriched in patients most likely to benefit from drugs that elevate renoprotective purines. Third, if clinical trials support the use of drugs that elevate renoprotective purines in COVID-19 patients, our results suggest that measurement of renoprotective purines in the urine could be used to guide therapy, an approach known as “precision medicine.”

Multiple organ failure (MOF) is common in severely ill COVID-19 patients. In our study, it seems that the severity of MOF (other than AKI) was similar in the AKI group versus the group without AKI (No AKI group). For example, 73.9% of the No AKI group versus 71.4% of the AKI group were in the ICU (*P* = 1). If MOF had been more severe/frequent in the AKI group, we would have anticipated that a higher percentage of the AKI group, compared to the No AKI group, would have been in the ICU. Also, if MOF had been more severe/frequent in the AKI group, we would have expected that a higher percentage of the AKI group, compared to the No AKI group, would have been on ECMO. However, none of the AKI patients were on ECMO, whereas 4 of 23 patients (17.3%) in the No AKI group were on ECMO (*P* = 0.12). This too would argue against higher levels of MOF in the AKI group. Finally, we believe the groups were fairly well matched with regard to MOF because the percentages of intubated patients in the two groups were similar and the groups were matched with regard to the WHO Ordinal Scale of Severity. Nonetheless, even though we could not detect a difference in MOF between the two groups, it is entirely possible that the AKI group did suffer from more damage to other organ systems. If so, the AKI group could also be considered a “MOF group.” In addition to the kidneys, it is known that adenosine, inosine and guanosine can protect other organ systems against damage^43,57–60^. Thus, our results may imply a deficiency of “organ protective” purines throughout the body. If so, drugs that elevate organ protective purines may offer multiple benefits to COVID-19 patients in addition to renal protection.

Our findings here, together with the findings of Correale et al., provide a sound rational for testing treatments that increase renoprotective purines in severely ill COVID-19 patients for protection against not only AKI but perhaps also MOF. The strong relationship between low levels of renoprotective purines in the urine and severity of AKI suggests that urine levels or urinary excretion rates of purines, particularly the sum of adenosine + inosine + guanosine, may provide a useful biomarker to guide patient selection in clinical trials to test the utility of appropriate purinergic therapies in COVID-19 patients.

## Data Availability

Data will be made available upon reasonable request to Dr. Edwin K. Jackson.
